# Perinatal factors for antepartum hemorrhage in women with placenta accreta spectrum

**DOI:** 10.3389/fsurg.2025.1588498

**Published:** 2025-06-25

**Authors:** Pengzhen Hu, Shaoxin Ye, Shuguang Zhou, Xiaoling Guo, Dazhi Fan

**Affiliations:** ^1^ The Second School of Clinical Medicine, Southern Medical University, Guangzhou, Guangdong, China; ^2^ Department of Obstetrics, The Affiliated Foshan Women and Children Hospital, Guangdong Medical University, Foshan, Guangdong, China; ^3^ Foshan Fetal Medicine Research Institute, the Affiliated Foshan Women and Children Hospital, Guangdong Medical University, Foshan, Guangdong, China; ^4^ Foshan Key Laboratory of Stem Cell and Maternal-Child Health, Foshan Women and Children Hospital, Foshan, Guangdong, China; ^5^ Stem Cell Clinical Research Center, Foshan Women and Children Hospital, Foshan, Guangdong, China; ^6^ Department of Gynecology, Maternal and Child Health Center of Anhui Medical University, The Fifth Affiliated Clinical College of Anhui Medical University, Hefei, Anhui, China

**Keywords:** placenta accreta spectrum, antepartum hemorrhage, placenta previa, risk factor, nomogram

## Abstract

**Introduction:**

Placenta accreta spectrum with antepartum hemorrhage is closely related to maternal and fetal morbidity and mortality. It is of utmost importance to predict the possibility of antepartum hemorrhage using perinatal factors before delivery in women with placenta accreta spectrum. The aim of this study is to identify the risk factors for antepartum hemorrhage in women with placenta accreta spectrum.

**Methods:**

This retrospective cohort study evaluated pregnant women with placenta accreta spectrum. Multivariate logistic regression was used to identify the independent variables associated with antepartum hemorrhage and a nomogram was developed to predict the possibility of antepartum hemorrhage. An Excel form computer interface was constructed to use the prediction model.

**Results:**

This retrospective cohort study included 188 participants (74 with antepartum hemorrhage). According to multivariate logistic regression analysis, blood type O [odds ratio (OR) 2.277, confidence interval (CI) 1.220–4.250], history of miscarriage (2.178, 95% CI 1.114–4.261), and parity (1.701, 1.037–2.790) were independent risk factors for antepartum hemorrhage in women with placenta accreta spectrum.

**Discussion:**

This results revealed that women with placenta accreta spectrum with blood type O, history of miscarriage, and multiparity may have a significant risk of experiencing antepartum hemorrhage.

## Introduction

Placenta accreta spectrum (PAS) is defined as abnormal placental trophoblast adherence with a varying range of myometrial invasion (1, 2). It is encompassed placenta accreta (chorionic villi attached to the myometrium), increta (villi present in the myometrium to the serosa), and percreta (villi penetrating the myometrium beyond the serosa) (3, 4). PAS is a serious pregnancy complication that can lead to severe postpartum hemorrhage, which may necessitate hysterectomy and can even cause maternal death (5–7). We previously reported that the prevalence of placenta accreta spectrum was about 0.2% among pregnant Chinese women (8). Due to the rise in global cesarean delivery rates, uterine surgery, advanced maternal age, and assisted reproductive technology, the incidence of placenta accreta spectrum has increased dramatically in recent years (9, 10).

Major obstetric hemorrhage in the antepartum setting can be life threatening and is a leading cause of intensive care unit admissions of pregnant women worldwide (11). Antepartum hemorrhage is bleeding in late pregnancy and can cause maternal and fetal complications, such as uteroplacental insufficiency, preterm birth, life-threatening maternal hemorrhage, and even perinatal mortality (12–14). In addition, antepartum bleeding is also associated with a greater risk of postpartum hemorrhage (15). Potential causes of severe antepartum hemorrhage include placental abruption, placenta previa, placenta accreta spectrum disorders, and vasa previa (11, 16). Previous studies have found that antepartum hemorrhage has an incidence of about 35% in women with placenta accreta spectrum (17, 18). In addition to well-known causes such as placental abruption and placenta previa along with their associated risk factors (9, 11), the aetiology and pathogenesis of antepartum hemorrhage in women with placenta accreta spectrum have not been clearly identified yet, and clinical studies focusing on placenta accreta spectrum in women with antepartum hemorrhage are rare.

Considering the dangers of antepartum hemorrhage resulting from placenta accreta spectrum, it is of utmost importance to prepare for the possibility of antepartum hemorrhage in women with placenta accreta spectrum based on perinatal factors. Unfortunately, a strategy to identify women at risk of antepartum hemorrhage has not been defined yet. This retrospective cohort aimed to investigate the risk factors for with antepartum hemorrhage among women with placenta accreta spectrum to enable the early prediction and timely management of antepartum hemorrhage in women with placenta accreta spectrum.

## Methods

This was a retrospective cohort analysis of participants who presented with placenta accreta spectrum at a tertiary care medical center between January 2012 and December 2022. The Foshan Women and Children Hospital Ethics Committee approved (FSFY-Med-2019044) access to participant information via electronic medical records and waived the requirement for informed consent.

The inclusion criteria were as follows: (1) gestational age between 28 and 42 weeks; (2) underwent prenatal imaging (ultrasound, MRI or cystoscopy) that raised suspicious of PAS; (3) and confirmed diagnosis of PAS after delivery (14, 19–21). Prenatal suspicion of PAS was based on clinical and imaging findings during pregnancy. The main ultrasound criteria used to suspect PAS included loss of the retroplacental sonolucent zone, presence of lacunae within the placenta, increased vascularity at the uterine-bladder interface, and abnormal placental location (e.g., low-lying or covering a previous cesarean scar) (14, 22). When ultrasound findings were inconclusive, MRI was used as an adjunctive tool, with T2-weighted images typically showing dark intraplacental bands, focal or irregular myometrial thinning, and marked heterogeneous placental enhancement (21). Cystoscopy was also occasionally performed in selected cases to rule out bladder invasion (14). The diagnosis of PAS after delivery was confirmed based on histopathological examination of the placental tissue combined with intraoperative findings (23, 24). Specifically, the presence of abnormal placental adherence to the myometrium without a clear plane of separation, or invasion beyond, was considered diagnostic of PAS (19, 20). The exclusion criteria were fetal malformation, intrauterine fetal death, coagulation dysfunction, and incomplete antenatal records. The participants were divided into placenta accreta spectrum with antepartum hemorrhage (PAS-APH) and placenta accreta spectrum without antepartum hemorrhage (PAS-non-APH) groups. Antepartum hemorrhage was defined as cumulative vaginal bleeding greater than 20 ml from 28 gestational weeks to prior to delivery (12, 13, 25).

Maternal characteristics, including maternal age at delivery, parity, preexisting diseases before pregnancy, prior dilatation and curettage, *in vitro* fertilization, regular antenatal care, gestational age, birth weight, sex of fetus, Apgar score at 5 min, risk factors associated with antepartum hemorrhage before 28 weeks of gestational age, and pregnancy complications, were analyzed. In this study, multiparity was defined as having had two or more previous deliveries beyond 28 weeks of gestation, whether those deliveries resulted in live births or stillbirths. Information of all participants obtained from the electronic medical records was anonymized prior to analysis in the form of an Excel file.

## Statistical analysis

Continuous data are reported as mean ± standard deviation, and count data are reported as rates (%). Multivariate logistic regression was used to determine the association of perinatal factors with risk of antepartum hemorrhage. Statistical analyses were performed using the R version 4.3.1 and SPSS 20. A *P*-value less than 0.05 was considered statistically significant. An Excel form computer interface was constructed to use the models.

## Results

After applying the exclusion criteria, 188 participants were included in this study. According to the definition of antepartum hemorrhage, 74 participants (74/188, 39.4%) were included in the PAS-APH group, and 114 participants (114/188, 60.6%) were included in the PAS-non-APH group. Table 1 shows the baseline characteristics of the participants. The PAS-APH group had a higher proportion of participants with blood type O (52.7% vs. 30.7%, *p* = 0.003), history of miscarriage (75.7% vs. 57.0%, *p* = 0.009), and male fetus (66.2% vs. 50.9%, *p* = 0.038) compared to the PAS-non-APH group. On performing multivariate logistic regression analysis, three perinatal factors were found to be independent predictors of PAS-APH: blood type O [odds ratio (OR) 2.277, confidence interval (CI) 1.220–4.250], history of miscarriage (2.178, 95% CI 1.114–4.261), and parity (1.701, 1.037–2.790) (Table 2).

**Table 1:** General characteristics of participants and multivariate logistic regression analyses for screening predictors.

Variables	APH (*n* = 74)	Non-APH (*n* = 114)	t/*χ^2^*/Z	*P*-value	OR (95% CI)		
Maternal age (years), mean ± sd	32.78 ± 4.87	33.21 ± 4.96	0.583	0.561	0.869	0.752	1.004
Advanced maternal age (>35 years old)	29 (39.2%)	42 (36.8%)	0.105	0.746	2.379	0.615	9.197
Maternal height (cm)	157.74 ± 5.30	156.63 ± 4.94	1.461	0.146	0.996	0.925	1.073
Blood type O	39 (52.7%)	35 (30.7%)	9.100	0.003	2.589	1.182	5.669
Married (%)	67 (90.5%)	110 (96.5%)	2.884	0.089	0.202	0.035	1.184
*in vitro* fertilization	1 (1.4%)	8 (7.0%)	3.161	0.075	0.336	0.024	4.730
Parity							
1	3 (4.1%)	16 (14.0%)	6.610	0.081	2.220	1.107	4.452
2	48 (64.9%)	75 (65.8%)					
3	20 (27.0%)	20 (17.5%)					
4	3 (4.1%)	3 (2.6%)					
Prior cesarean delivery	62 (83.8%)	90 (78.9%)	0.678	0.453	0.508	0.173	1.490
History of vaginal delivery	14 (18.9%)	20 (17.5%)	0.057	0.811	1.097	0.515	2.335
History of miscarriages (%)	56 (75.7%)	65 (57.0%)	6.810	0.009	2.396	1.028	5.585
Twins	2 (2.7%)	6 (5.3%)	0.722	0.395	0.106	0.007	1.639
Anterior placenta	46 (75.4%)	68 (73.1%)	0.101	0.751	1.066	0.427	2.661
Complete placenta previa	60 (81.1%)	81 (71.1%)	2.407	0.121	1.534	0.592	3.977
PAS							
PA	46 (62.2%)	58 (50.9%)	3.473	0.176	0.616	0.361	1.052
PP	27 (36.5%)	50 (43.9%)					
PI	1 (1.4%)	6 (5.3%)					
Male-fetus	49 (66.2%)	58 (50.9%)	4.305	0.038	2.155	0.961	4.835

PA, placenta accreta; PAS, placenta accreta spectrum; PI, placenta increta; PP, placenta percreta.

**Table 2:** The logistic regression of five perinatal factors for constructing model.

Variables	*β*	SE	Wald	*P*-value	OR (95% CI)		
Maternal age	−0.028	0.033	0.727	0.394	0.972	0.910	1.038
Blood type O	0.823	0.318	6.680	0.010	2.277	1.220	4.250
History of miscarriages	0.779	0.342	5.172	0.023	2.178	1.114	4.261
Parity	0.531	0.252	4.432	0.035	1.701	1.037	2.790
Constant term	−1.524	1.176	1.679	0.195	0.218	-	-

SE, standard error; OR, odds ratio; CI, confidence interval.

**Figure 1:**
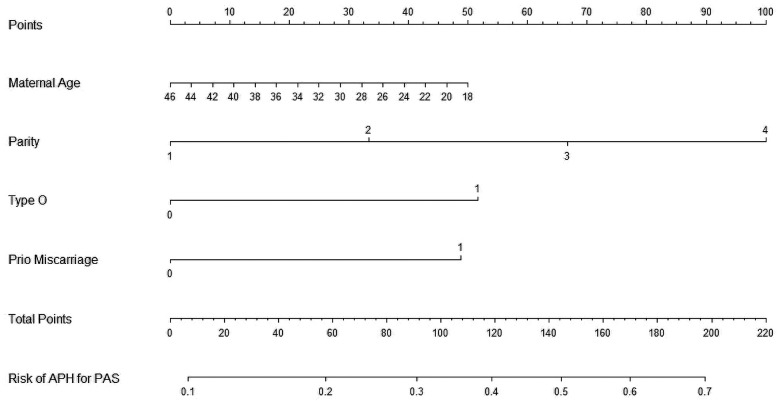
Nomogram for the antepartum hemorrhage for placenta accreta spectrum women. APH, antepartum hemorrhage; PAS, placenta accreta spectrum.

**Figure 2:**
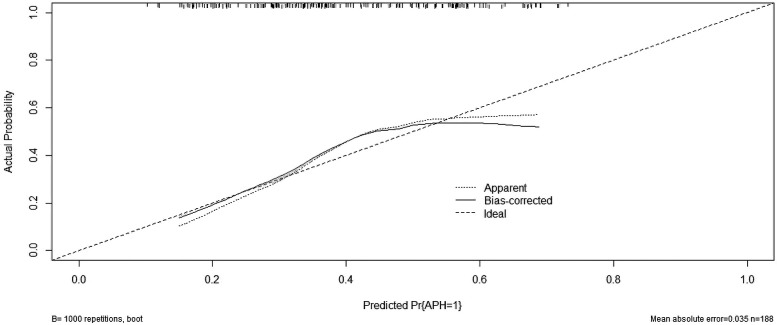
Calibration curve for predicting probability of antepartum hemorrhage for placenta accreta spectrum women. APH, antepartum hemorrhage; PP, placenta accreta spectrum.

**Figure 3:**
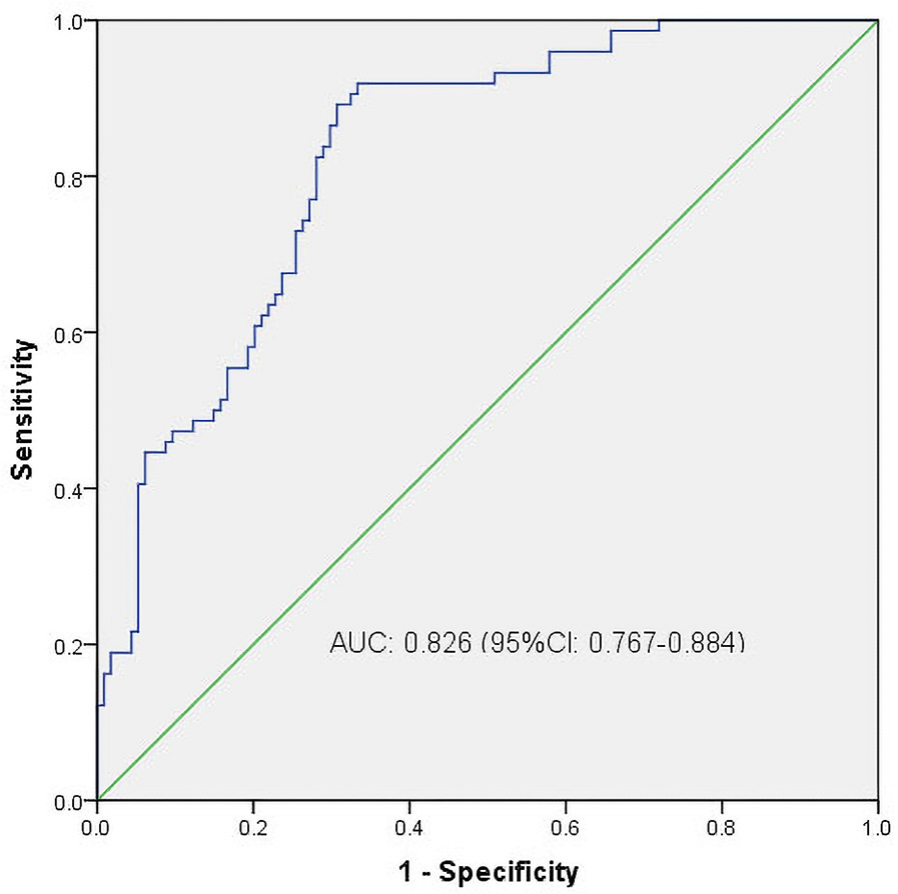
ROC curves. ROC, receiver operating characteristic; AUC, area under the ROC curve; CI, confidence interval.

**Figure 4:**
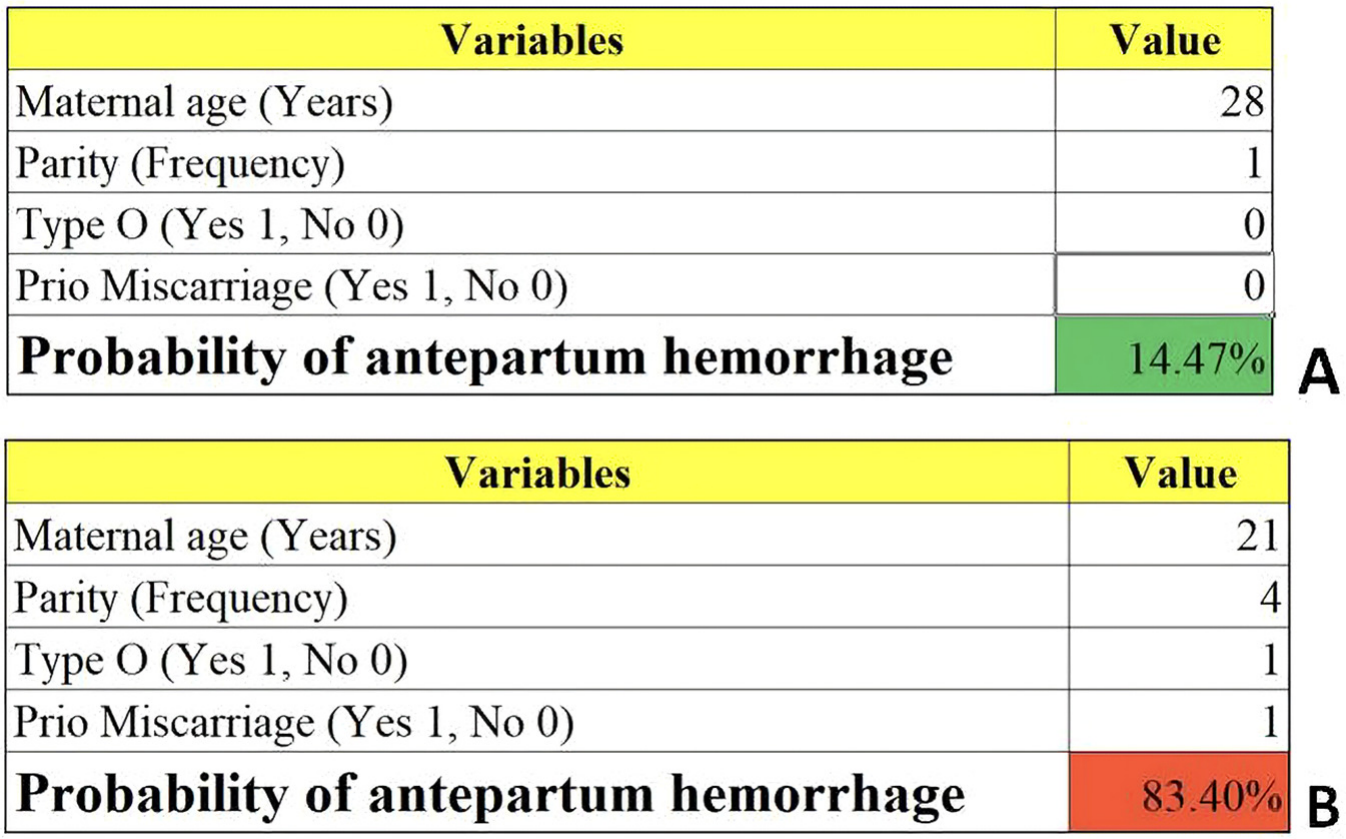
Examples of the computer interface (excel form). Two examples of placenta accreta spectrum women with low **(A)** and high risk **(B)** of antepartum hemorrhage.

We then constructed a nomogram by comabining the factors obtained from the logistic regression analysis (blood type O, history of miscarriage, parity) and maternal age (Figure 1). Each factor was assigned a point value, and the total score matched the predicted probability according to their contribution to the overall antepartum hemorrhage risk in women with placenta accreta spectrum. The bias-corrected curve was statistically close to the ideal cureve, and the nomogram calibration curves are shown in Figure 2. The receiver operating characteristic (ROC) cure of the nomogram is shown in Figure 3. It had an area under the curve (AUC) value of 0.826 (95% CI: 0.767–0.884). Additionally, we constructed an Excel form computer interface to calculate the likelihood of antepartum hemorrhage in pregnant women with placenta accreta spectrum using the aforementioned prediction model. The interactive interface generated based on Excel can be viewed in the attachment. An example of this interface is shown in Figure 4.

## Discussion

In this study, we found that blood type O, history of miscarriage, and parity increased the risk of antepartum hemorrhage in women with placenta accreta spectrum. We also developed and validated a nomogram based on four perinatal factors (blood type O, history of miscarriage, parity, and maternal age) that accurately predicted the probability of antepartum hemorrhage in women with placenta accreta spectrum.

ABO blood types are the most important blood groups in clinical practice and their relevance to reproductive well-being has been studied (26–29). Franchini et al. described an association between blood type O and venous thromboembolism in pregnant women (30). A retrospective study demonstrated that women with blood type B had an increased risk of gestational diabetes mellitus compared to women with blood type O during singleton pregnancies, while blood type A was associated with a higher risk of placenta previa during twin pregnancies (31). Our recently study on women with placenta previa found that the incidence of antepartum hemorrhage, predelivery anemia, and preterm birth was higher in women with blood type AB, and the incidence of neonatal asphyxia was higher in those with blood type O (32). In this study, we found that the risk of antepartum hemorrhage was increased by 2.2-fold in women with placenta accreta spectrum women with blood type O compared to that in women with blood type non-O blood types. As an independent predictor, blood type O increases the likelihood of antepartum hemorrhage in women with placenta accreta spectrum according to the results of our study.

While our study did not directly explore these mechanisms, existing literature provides some clues that might explain this association. Firstly, individuals with blood type O have lower levels of von Willebrand factor (vWF) and factor VIII compared to other blood types, which could contribute to impaired hemostasis and thus increase the risk of bleeding disorders, including conditions like APH in pregnant women with PAS (28, 32). Secondly, ABO blood group antigens are expressed on the surface of various cells, including endothelial cells, potentially influencing cellular interactions and adhesion processes that are crucial for placentation (29, 31). In the context of PAS, where abnormal placental attachment is a hallmark, differences in how blood type O affects these processes could lead to variations in the risk of developing severe bleeding complications. Lastly, there is evidence suggesting that blood type O is associated with elevated levels of soluble fms-like tyrosine kinase-1 (sFlt-1), a protein linked to the pathophysiology of preeclampsia and other placental-related disorders (27, 30). Elevated sFlt-1 levels could disrupt angiogenic balance, potentially contributing to abnormal placental development and increasing the risk of APH in PAS cases.

A history of miscarriage is associated with various maternal and neonatal complications, which have been reported in the literature. The most common complications consistently linked to a history of miscarriage are preterm birth, preeclampsia, low birth weight, and increased perinatal mortality (33). A systematic review and meta-analysis showed that women with a history of miscarriage are at increased risk of preterm birth in subsequent pregnancies (34). Similarly, a retrospective cohort study conducted over 10 years found that there was an increased risk of adverse obstetric and perinatal outcomes, including placental dysfunction disorder (preterm preeclampsia and preterm birth), and abnormal placentation (placenta previa and placenta accrete), in a subsequent pregnancy among women with a history of miscarriage (35). The present study demonstrates an association between a history of miscarriage and increased risk of antepartum hemorrhage in women with placenta accreta spectrum. This finding may be useful in guiding the clinical management of placenta accreta spectrum in women with a history of miscarriage.

Although parity has been reported to be associated with maternal and neonatal outcomes, the results were inconsistent. A study has claimed that the prevalence of postpartum bleeding, postpartum anemia, gestational diabetes, and preterm pregnancy is related to multiparity (36). In contrast, other studies have reported that multiparous women have similar risks of maternal and neonatal complications as the other parity groups (37). We previously showed a positive correlation between multiparity and the prevalence of antepartum hemorrhage in a meta-analysis that included 29 articles (25). However, Nur Azurah et al. Revealed that there was no difference in the incidence of antepartum hemorrhage between 56 primigravida and 187 non-primigravida women (38). Other factors, such as socioeconomic and sociocultural factors, might play a major role in determining pregnancy outcomes. Because multiparity is more likely to occur in older women, the risks might also be attributed to advanced maternal age in addition to high parity. Interestingly, we found that multiparity was a significant risk factors for antepartum hemorrhage in women with placenta accreta spectrum after adjusting for maternal age.

Maternal age at birth is increasing globally, and is associated with perinatal outcomes (39). A prospective multicenter cohort study found that maternal age is nonlinearly associated with a greater risk of placenta accreta spectrum, placenta previa, gestational diabetes mellitus, hypertensive disorders of pregnancy, preeclampsia, cesarean delivery, preterm birth, large for gestational age, macrosomia, and fetal congenital anomaly, with inflection points around 35.6–40.4 years, but is not related to postpartum hemorrhage or small for gestational age (40). Kuribayashi et al. showed that maternal age below 30 years is more likely to be associated with antepartum hemorrhage in an individual pregnant woman with placenta previa (41). However, studies on the risk of antepartum hemorrhage in women with placenta accreta spectrum women are sparse and equivocal. We did not identify an association between maternal age and antepartum hemorrhage in women with placenta accreta spectrum using multivariate logistic regression analysis. Considering the effects of maternal age on perinatal outcomes, maternal age was considered when constructing the nomogram.

While maternal age did not emerge as a significant factor in this specific context, we believe its inclusion in the nomogram remains justified for several reasons. Firstly, maternal age is widely recognized for its broad impact on perinatal outcomes, making it a clinically relevant factor even if not statistically significant in our model. Secondly, the nomogram serves as a practical tool designed to integrate multiple risk factors, some of which may influence risk through complex interactions or subtle effects that are not fully captured by statistical significance alone. Additionally, maternal age might interact with other variables such as parity or prior cesarean sections, potentially influencing APH risk in ways not fully explored in our initial analysis. Lastly, including maternal age enhances the interpretability and educational value of the nomogram for clinical practitioners who routinely consider maternal age when assessing obstetric risks.

We evaluated the prenatal risk factors for antepartum hemorrhage in women with placenta accreta spectrum and developed a likelihood prediction model for the early identification and management of antepartum hemorrhage in women with placenta accreta spectrum. Our model demonstrated good accuracy and conformity. As a visual and individualized model, a nomogram is an easy-to-use and understandable tool for realistic predictions in clinical practice. However, it needs to be emphasized is that while nomogram is an advantageous and valuable tool for making decisions, clinical judgment remains central to decision-making. While we fully recognize that PAS is associated with a high baseline risk of bleeding, our objective in identifying additional perinatal factors was not to replace current management protocols, but rather to explore potential variables associated with antepartum hemorrhage in this population. We believe such findings may support more refined risk stratification, enhance antenatal counseling, and inform individualized care strategies.

To the best of our knowledge, this is the first study to propose and verify a straight forward technique for estimating the risk of antepartum hemorrhage in women with placenta accreta spectrum in a large group of individuals. However, this study has several limitations that need to be noted. First, recall bias was inevitable, and may have influenced the results because of the retrospective nature of the analysis. Second, owing to the samll sample size and lack of data, several potentially meaningful predictors, including cervical length during pregnancy (before an attack of antepartum bleeding), history of bleeding in the first trimester, previous lower segment cesarean section and placenta previa, weight gain during pregnancy, and maternal occupation, were not evaluated in this study (42, 43). Last, although our study was conducted at a single tertiary care center, the diverse patient population and standardized clinical protocols suggest that our findings may be applicable to similar urban medical centers managing complex obstetric cases. However, differences in healthcare practices, resource availability, and prenatal care access across regions may affect the generalizability of our results. Future prospective multi-center large-sample studies are still needed to further verify the stability and representativeness of the results of this research. Additional potential indicators need to be evaluated in conjunction with clinical variables in a subsequent study to establish a more precise forecasting algorithm for antepartum hemorrhage with the aim of reducing maternal and newborn mortality.

## Conclusions

Overall, this study revealed that women with placenta accreta spectrum with blood type O, history of miscarriage, and multiparity may have a significant risk of experiencing antepartum hemorrhage. We also developed the first perinatal prediction nomogram based on several perinatal factors for the early prediction of antepartum hemorrhage in women with placenta accreta spectrum. Our prediction score may prove to be a valuable resource for doctors, midwives, and obstetric nurses, and the nomogram may provide assistance in clinical decision-making.

## Data Availability

The raw data supporting the conclusions of this article will be made available by the authors, without undue reservation.

## Supplementary material

Supplementary File 1 Computer interface (Excel form) of antepartum hemorrhage for placenta previa women.
